# Trends in Health Equity Among Children in the United States, 1997–2018

**DOI:** 10.1007/s10995-021-03253-w

**Published:** 2021-10-15

**Authors:** Nathaniel W. Anderson, Frederick J. Zimmerman

**Affiliations:** Present Address: Department of Health Policy and Management, University of California Los Angeles, 650 Charles E Young Dr S, Los Angeles, CA 90095 USA

**Keywords:** Health equity, Child health, Health status disparities, Public health surveillance

## Abstract

**Objectives:**

Health equity is crucial to population health. To achieve this aim, extensive monitoring efforts beyond traditional disparities research are required. This analysis assesses trends in health equity for children from 1997 to 2018.

**Methods:**

Health equity in a given year is calculated using a previously developed measure as the mean weighted departure of individual health from the best achievable level of health. This criterion is defined as the median health of the most socially privileged identifiable group: white, non-Latinx boys in upper-income households.

Using more than 20 years of data from the National Health Interview Survey, we apply this methodology to six measures of child health: parent-reported health status, school days missed due to illness or injury in the past year, a strength and difficulties questionnaire score, emotional difficulties, a toddler mental health indicator score, and toddler depression. We separately calculate racial/ethnic and income disparities.

Monte Carlo simulation is used to assess whether trends are statistically significant.

**Results:**

Health equity among children increased gradually over the past 2 decades, with five of the six measures demonstrating upward trends. Improvements in health equity are stronger among younger children (age 0–3 and 4–7). Unlike previous work examining adults, both types of disparities narrowed over the study period.

**Conclusions for Practice:**

Progress on health equity requires accountability to an objective metric. This analysis suggests some improvement over the past two decades, although these gains are under threat from potential decreases in government spending on programs affecting children and the COVID-19 pandemic.

## Significance Statement

*What is Already Known on This Subject* Previous analyses have documented declines in health disparities among children. However, progress in health equity does not necessarily occur under these conditions. As an example, previous work has found that health equity for adults declined in the past 20 years despite narrowing racial/ethnic disparities.

*What This Study Adds* Applying a metric of health equity to children for the first time, we observe gradual increase in health equity over the past 20 years, with gains concentrated in younger children.

## Introduction

In recent years, leaders in public health have placed greater emphasis on achieving equity in population health (Healthy People 2030, 2020; Plough, 2015). While health equity has multiple meanings in the literature, Braveman and colleagues have carefully conceptualized health equity as the conditions under which “everyone has a fair and just opportunity to be as healthy as possible” (Braveman, 2017). Occasionally, “as healthy as possible” is also referred to as individuals reaching their “health potential” (Whitehead, 1991).

One of the roles of public health is to conduct rigorous monitoring and surveillance of populations. This practice is particularly essential for achieving health equity. Traditionally, public health officials use measures of health disparities—that is, the difference in average health between distinct populations—as the metric to evaluate progress toward health equity (Braveman, 2014). However, relying solely on disparity indicators can be problematic. Focusing on differences across a single dimension (i.e. race/ethnicity or sex) ignores the importance of intersectionality across these groups. The health disparities experienced by Black women cannot be reduced to the sum of disparities experienced by Black men and the disparities experienced by white women. Similarly, measures of disparities can oversimplify the experiences of diverse populations that may face unique challenges, such as immigrant or religious subpopulations. Weaknesses of the disparities approach are especially concerning for smaller, more marginalized groups that are not acknowledged in current population health surveillance, such as religious or sexual minorities. Grave injustices experienced by marginalized groups are missed when these persons are lumped into larger subpopulations corresponding to other aspects of their identity. As such, disparity measures may not always be well-suited to monitoring population-level health equity.

To continue tracking overall progress towards achieving health equity, while addressing some of these issues, previous research has developed a population-based measure that aligns better with the conceptualized goals of health equity (Zimmerman, 2019). This Health Equity Metric (HEM) has documented an overall declining trend in health equity among the adult population in the United States between 1993 and 2017, despite improvements in racial/ethnic disparities over the same period (Zimmerman & Anderson, 2019).

However, this methodology has not been applied to children and adolescents. Addressing this gap in knowledge is important for several reasons. Per-capita federal spending on children has increased by nearly 75% since 2000, with the majority of the increased spending specifically going towards health and nutrition programs for lower-income families (i.e. Medicaid, CHIP, and SNAP) (Isaacs et al., 2019). Furthermore, inequalities in infant and child mortality have narrowed since the 1990s (Currie & Schwandt, 2016). Thus, health equity may have increased among children at the same time it declined among adults. Furthermore, a large body of research has demonstrated that inequities in childhood result in lifelong disparities (Kuh & Shlomo, 2004). Therefore, examining health-equity trends in the younger population may shed light on why progress among adults has stalled in recent years. Lastly, decomposing trends within the child population by age group or geographic region could suggest critical areas for policymakers and researchers to direct their attention.

Using data from a large population-health data source, we apply the health equity metric, along with several disparities-based measures, to a series of health indicators to assess trends among children and adolescents (age 0–17) from 1997 to 2018.

## Methods

### Data Source

This study follows the STROBE guidelines for cross-sectional studies and does not qualify as human subjects research (von Elm et al., 2014). Data are from the National Health Interview Survey (NHIS), through the Integrated Public Use Microdata Series NHIS database (Blewett et al., 2019). The NHIS is a repeated cross-sectional survey that allows for nationally representative estimates of population health. The child supplement randomly selects one child (0–17) in each family and asks the respondent—typically a parent—an additional set of questions about the child’s general and mental health and other related outcomes. Information is collected for approximately 8000–14,000 children in each year during the study period.

The health equity metric requires measures of health that are continuous or reasonably semi-continuous. The following measures are selected from the sample child questionnaire and available for all years unless otherwise noted:

- A measure of parent-reported general health status, reweighted to approximate a semi-continuous variable using results from a previous study (Van Doorslaer & Jones, 2003). The estimated health weights are: 0.983 = excellent, 0.931 = very good, 0.841 = good, 0.707 = fair, 0.401 = poor.
- Days of school missed in the past year due to illness or injury, ages 5–17 (top-coded at 40).
- Strength and Difficulties abbreviated questionnaire (SDQ), ages 4–17; 2001–2007 and 2010–2018. The scale ranges from 0 to 10, based on the sum of responses that are “certainly true” (2 points), “somewhat true” (1 point), or “not true” (0 points) to each of the following prompts: gets along better with adults than peers; good attention span and finishes tasks; often unhappy, depressed, or tearful; generally well-behaved; often seems worried.
- Emotional difficulties, ages 4–17; 2001–2018, whether the child had “severe difficulties” (3 points), “definite difficulties” (2 points), “minor difficulties” (1 point), or “No” (0 points) with emotions, concentration, behavior, or being able to get along with other people.
- Toddler Mental Health Indicator (MHI) Score, ages 2–3. The scale ranges from 0 to 8 based on the sum of responses that are “not true” (0), “sometimes true” (1), or “often true” (2) to each of the following prompts: how often uncooperative; how often has trouble getting to sleep; has speech problems; has been unhappy, sad, or depressed for the past 2 months.
- Toddler Depression, ages 2–3; responded “not true” (1 point), “sometimes true” (2 points), or “often true” (3 points), to a prompt about whether child had been unhappy, sad, or depressed in the past 2 months.

Measures are reverse-coded such that positive outcomes had higher values when calculating the health equity metric.

The measures used in this analysis encapsulate the multidimensional nature of health. They span the three domains of children’s health articulated by the Institute of Medicine in its 2004 report “Children’s Health, the Nation’s Wealth: Assessing and Improving Child Health”: health conditions, functioning, and health potential (National Research Council; Institute of Medicine, 2004). Both physical and mental health are represented. Some measures focus on early childhood and adolescence, while others span the entire 0–17 population to allow for comparisons across developmental stages. As a prerequisite for inclusion on the NHIS, these measures have been extensively validated in prior research, including across a range of social contexts (Achenbach & Rescorla, 2001; Ebesutani et al., 2010; Goodman, 1997, 2001; Goodman & Goodman, 2011; Newacheck et al., 1991; Seligman et al., 2004).

### Defining the Health Equity Metric

The health equity metric is calculated as the average deficit of individual health from a benchmark level of health, conceptualized as the overall “health potential” for a given population. If all members of a population have a level of health at this value, true health equity has been realized.

**Fig. 1:**
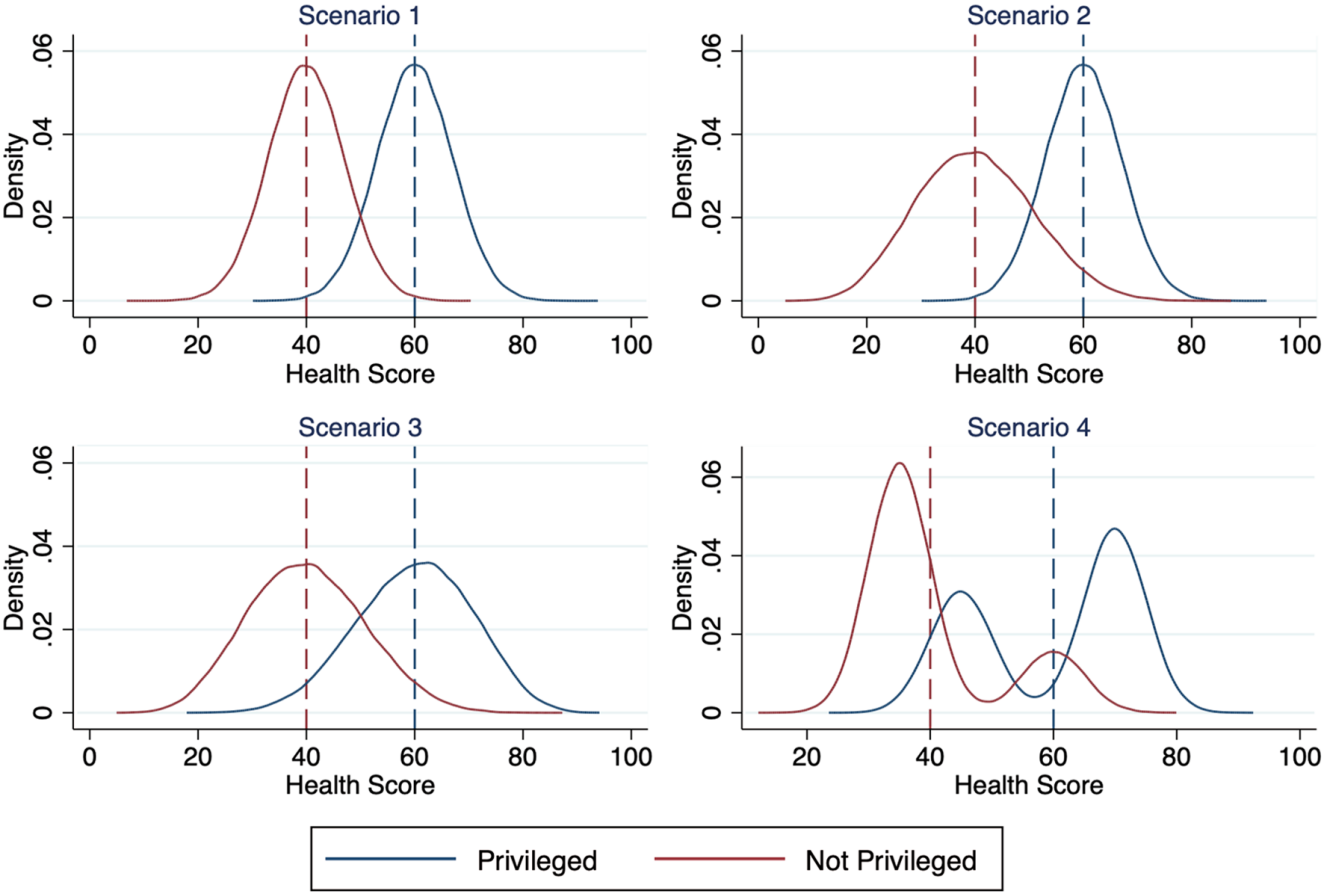
Scenarios where same health disparities have different health equities. For each scenario, we simulate a health outcome ranging from 0 to 100 for 100,000 privileged and 100,000 non-privileged persons. Each graph plots the probability density function of the two populations. In all scenarios, the mean value for the privileged group is 60 and the mean value for the non-privileged group is 40 (each represented by the corresponding dashed lines), meaning the health disparity is always a value of 20. However, the HEM declines for each successive scenario shown. Simulations are performed in STATA

Larger individual deficits from this benchmark level have greater weight than multiple smaller deficits summing to the same total deficit. This attribute is critical for understanding how health equity and health disparities measures, though correlated, are distinct. Figure 1 provides an illustrative example. For simplicity, we assume an individual’s health is expressed as a number between 0 and 100, and plot several hypothetical health distributions of two equal-sized groups, one privileged and the other non-privileged. In each scenario, health disparities are identical: the dashed lines showing average health for each subpopulation remain at the same value. At the same time, each successive scenario exhibits progressively less overall health equity.

Each successive scenario also represents a different manner in which health equity could decline. In moving from the first to the second scenario, the health distribution of the non-privileged group widens, reflecting greater inequality within the marginalized group. Moving from the second to the third scenario, the health distribution of the *privileged* group widens. In both Scenarios 2 and 3, portions of the population move further from the benchmark level of health (represented by the privileged group’s dashed line), relative to Scenario 1. Because health equity emphasizes the ability of *all* persons to reach their health potential, and because larger departures incur a proportionately larger decrement to health equity, each of these distributional shifts corresponds to a lower health equity metric, with that of Scenario 3 being even lower than that of Scenario 2.

The last scenario illustrates how health disparities measures are not well-suited to deal with marginalization across multiple dimensions. In this example, the privileged and non-privileged populations each contain subgroups with their own distributions, reflecting the important role of intersectionality. As an example, they might reflect educational, income, religious, or gender health gradients existing within racial/ethnic disparities. A health-disparities measure would report that the fourth scenario is identical to the first, yet the last scenario exhibits a stark form of inequality. Rather than assuming that the subpopulation mean adequately represents the experience of the whole subpopulation, the HEM provides information on the total population, including systematic deficiencies in the health of smaller marginalized groups. Whereas health disparities tend to focus on a singular form of marginalization, the HEM is sensitive to all forms of marginalization.

The formula for the HEM is as follows:

$$HEM=1-\frac{1}{N}\sum_{i=1}^{N}{\left(\beta \times \mathrm{max}\left\{\frac{{\overline{y} }^{*}-{y}_{i}}{{\overline{y} }^{*}},0\right\}\right)}^{\alpha }$$

where HEM is the health equity metric, N is the total number of individuals in the sample (both privileged and non-privileged), y_i_ is individual *i*'s health status, y̅* is the benchmark level of health, and α and β are scale parameters that ensure larger deficits from the benchmark level of health are weighted more heavily. Consistent with prior literature, we use values of α = 2.5 and β = 2 (Zimmerman, 2019). To make the measure more easily interpretable, we multiply each estimate by 1,000. Since equity is concerned with populations reaching their health potential, individuals with levels of health equal to or greater than the benchmark level are assigned the same maximum contribution to the HEM. The range for the HEM is 1000 × (1−β^α^) (maximum health inequity) to 1000 (true health equity).

Because health equity calls attention to the social, economic, and political forces that shape our health, we conceptualize the population’s health potential as the median health of the most socially-privileged identifiable subgroup. We opt for white, non-Latinx boys with household income greater than 400% FPL, since relative to their peers they would experience the fewest structural disadvantages and barriers that might affect their health. However, various alternatives exist for the benchmark level of optimal health. To ensure that the findings are robust to this decision, we also estimated the HEM using the 90% percentile among the entire population as the measure of health potential, the results of which were not meaningfully different from our primary specification (see Appendix Figs. 5 and 6).

### Additional Measures

Changes in the health equity metric can be the result of various sub-trends (Zimmerman & Anderson, 2019). Different sub-trends, such as the privileged group worsening or other groups improving, could have notably different policy implications. Therefore, we calculate Black–white, Latinx-white, and income disparities to better understand the underlying drivers in trends of health equity. Income-related disparities are estimated as the difference in means between children in families with income < 150% of the federal poverty line (FPL) and children in families with incomes ≥ 400% FPL. The family-income thresholds approximately divide the child population into thirds over the study period.

### Analytic Strategy

All analyses are performed in STATA version 16, accommodating the complex survey design of the NHIS and using multiple imputation files for household income. The following age-stratified analyses are conducted: 0–3 years (general health); 4–7 years (general health, school-loss days, SDQ score, and emotional difficulties); 8–12 years (general health, school-loss days, SDQ score, and emotional difficulties); and 13–17 years (general health, school-loss days, SDQ score, and emotional difficulties). We also conducted a sub-analysis by Census region, but did not identify any noticeable differences in trends across geographic level (Appendix Figure 7).

Each annual estimate of the HEM is presented with its 95% CI. To assess whether trends in the HEM and disparity measures are statistically significant, we conduct a Monte Carlo simulation with 10,000 replications. For each iteration, values for annual estimates are drawn from a normal distribution, using the mean and variance parameters from the original estimation. A trend line is constructed from these draws and a 95% credible interval of the trend is computed as the 2.5th percentile and 97.5th percentile of the resulting 10,000 estimated slopes of the trend.

## Results

Table 1 shows descriptive statistics of the sample for select years. In general, the child population remains relatively consistent in demographic and socioeconomic makeup from 1997 to 2018. The proportion of the population considered privileged—upper-income white, non-Latinx males—is relatively stable over time: between 9.7 and 12.4 percent in any given year. Average health outcomes are either improving (SDQ score, toddler depression, school-loss days) or relatively stable (general health status, emotional difficulties, toddler mental health score).

**Table 1:** Descriptive characteristics of the sample child sample, select years

	1997	2001	2005	2009	2013	2017
Age						
0–3	22.0%	21.8%	22.2%	22.9%	21.2%	21.1%
4–7	23.0%	21.9%	21.0%	22.0%	22.8%	22.5%
8–12	27.9%	28.5%	27.9%	27.3%	28.1%	28.3%
13–17	27.2%	27.7%	28.9%	27.7%	27.9%	28.1%
Sex						
Male	51.2%	51.1%	51.1%	51.1%	51.1%	51.0%
Female	48.8%	48.9%	48.9%	48.9%	48.9%	49.0%
Race/ethnicity						
White, non-latinx	65.2%	63.5%	59.9%	56.6%	54.4%	52.8%
Black, non-latinx	15.0%	14.8%	15.1%	15.1%	14.7%	14.5%
Latinx	15.0%	16.8%	19.7%	22.3%	24.2%	25.2%
Other/multiple, non-latinx	4.8%	5.0%	5.3%	6.0%	6.7%	7.5%
Household income						
Less than 150% FPL	31.2%	28.7%	29.7%	33.6%	35.1%	30.1%
150–399% FPL	44.9%	41.9%	42.1%	40.1%	38.6%	39.4%
400% FPL or Greater	23.9%	29.4%	28.2%	26.3%	26.3%	30.5%
Privileged (Male; White, non-latinx; 400% FPL or greater)	9.7%	12.4%	11.7%	10.4%	9.7%	11.2%
Reweighted health status^a^	0.940 (0.0007)	0.942 (0.0006)	0.940 (0.0008)	0.943 (0.0008)	0.943 (0.0009)	0.946 (0.0010)
SDQ questionnaire^b^		8.01 (0.022)	8.32 (0.022)		8.44 (0.022)	8.42 (0.028)
Emotional difficulties^c^		2.71 (0.007)	2.74 (0.007)	2.74 (0.009)	2.75 (0.007)	2.72 (0.009)
Toddler MHI score^b^	6.72 (0.047)	6.65 (0.050)	6.68 (0.044)	6.79 (0.052)	6.74 (0.052)	6.66 (0.062)
Toddler depression score^b^	1.84 (0.013)	1.85 (0.013)	1.89 (0.010)	1.91 (0.011)	1.89 (0.014)	1.91 (0.013)
Days of school missed in past year^b, d^	36.27 (0.059)	36.35 (0.068)	36.72 (0.058)	36.55 (0.071)	36.85 (0.065)	36.93 (0.068)
N	14,290	13,579	12,523	11,156	12,860	8,845

Standard errors for averages are included in parentheses. SDQ score is not assessed in 1997 or 2009, and emotional difficulties is not assessed in 1997. For health status variables, results are presented such that higher values indicate a positive health outcome. More detail is provided in item-specific notes below

SDQ Strengths and Difficulties Questionnaire, MHI Mental Health Indicator

^a^Values for scoring average are taken from Van Doorslaer and Jones and are as follows 0.983 = excellent, 0.931 = very good, 0.841 = good, 0.707 = fair, 0.401 = poor)

^b^Values for scoring average reverse-coded from original scale score

^c^Values for scoring average are as follows: 3 = No, 2 = yes, minor difficulties, 1 = yes, definite difficulties, 0 = yes, severe difficulties

^d^Maximum number of days missed is top-coded at 40

**Fig. 2:**
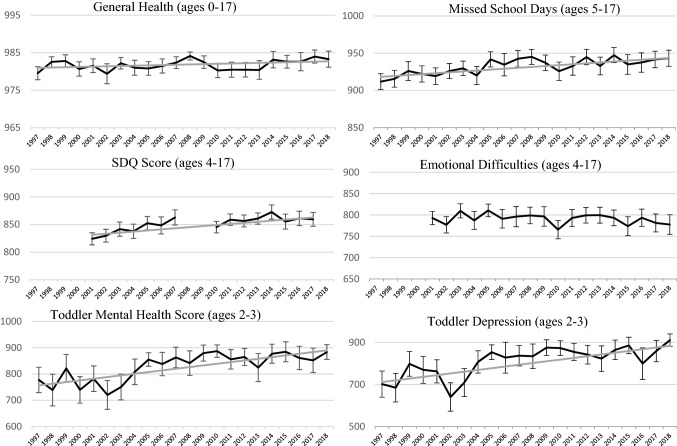
Health equity metric, selected child health indictors 1997–2018. Source: 1997–2018 National Health Interview Survey, Sample Child Questionnaire. *HEM* Health Equity Metric. *SDQ* Strength and Difficulties Questionnaire. Age of respondents is shown in parentheses. The graphs display the value of the HEM for the entire child population over the study period for the various measures. HEM is rescaled by 1000 to assist with interpretability—a value of 1000 indicates perfect health equity. Response values in reweighted general health are based on the results from Van Doorslaer & Jones, 2003. 95% CI for the health equity metric estimates are denoted by hashes. Omitted points reflect the question is not included in the survey for that year. The median trend from Monte Carlo simulation is shown in gray if the 95% credible interval indicates the trend is significantly different from 0

Figure 2 shows the estimated health equity metric for each outcome, along with the median simulated trend, provided the 95% credible interval did not include the null; otherwise the trend line is omitted. Despite relatively consistent averages in health outcomes over time, health equity has increased from 1997 to 2018. Across the six measures, the HEM tends to exhibit an upward trend, with all but one of the credible intervals not including zero. For general health, the upward trend is slight (0.08; 95% CI 0.02, 0.15). Toddler mental health measures show the strongest trend [(toddler MHI: 6.33; 95% CI 4.81, 7.89); (toddler depression: 8.22; 95% CI 6.34, 10.12)]. Trends in equity for SDQ score and school-days missed are moderate [(SDQ: 1.97; 95% CI 1.35, 2.58); (school-days missed: 1.21; 95% CI 0.83, 1.59)].

**Fig. 3:**
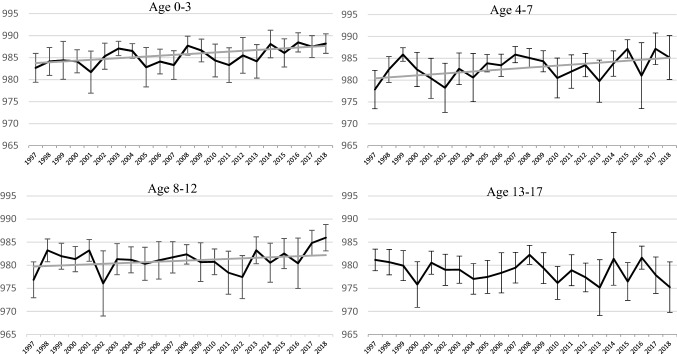
Health equity metric, reweighted parent-reported health, by age geoup: 1997–2018. 1997–2018 National Health Interview Survey, Sample Child Questionnaire. *HEM* Health Equity Metric. The graphs display the value of the HEM for the child population by age subgroup over the study period. HEM is rescaled by 1000 to assist with interpretability—a value of 1000 indicates perfect health equity. Response values in reweighted general health are based on the results from Van Doorslaer & Jones, 2003. 95% CI for the health equity metric estimates are denoted by hashes. The median trend from Monte Carlo simulation is shown in gray if the 95% credible interval indicates the trend is significantly different from 0

Figure 3 shows age-stratified HEMs for general health status. All age groups have generally similar levels of equity at the beginning of the study period: HEM values range from 977 to 983 and an ANOVA test does not find that at least one of the subgroup means is statistically different from the others at the 0.05 level (F-statistic = 2.3928; p-value = 0.0665). Increases in health equity are evident for the younger age groups [(age 0–3: 0.19; 95% CI 0.08, 0.29); (age 4–7: 0.22; 95% CI 0.18, 0.28)]. For older children, the trend in the HEM is negative but not statistically significant. For the measures besides general health status, the findings are similar: improvements in the HEM are typically strongest among younger children, with the one exception being school-loss days (Appendix Figs. 8, 9, 10).

**Fig. 4:**
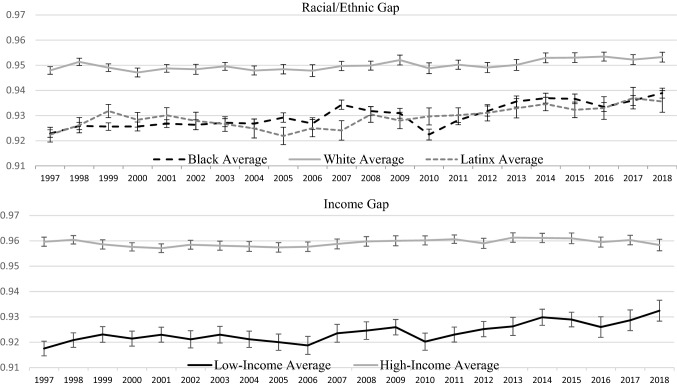
Disparities measures, reweighted parent-reported health: 1997–2018. Source: 1997–2018 National Health Interview Survey, Sample Child Questionnaire. The trends here show the average value of the parent-reported health measure for respective subpopulations. Response values in reweighted general health are based on the results from Van Doorslaer & Jones, 2003. Low-income and high-income are defined as living in a household with income < 150% of the federal poverty line and living in a household with income ≥ 400% of the federal poverty line, respectively. 95% CI for the annual estimates are denoted by hashes. Monte Carlo simulation estimates show that the Black–white gap (− 0.00039; 95% CI: − 0.00023, − 0.00055), the Latinx-white gap (− 0.00023; 95% CI: − 0.00010, − 0.00036) and the income gap (− 0.00037; 95% CI: − 0.00024, − 0.00050) narrowed over the study period

Figure 4 shows racial/ethnic disparities and income disparities for general health status. Monte Carlo simulation estimates find that Black–white disparities (− 0.00039; 95% CI − 0.00023, − 0.00055) and income disparities (− 0.00037; 95% CI − 0.00024, − 0.00050) narrowed over the study period by approximately the same amount, while the decline in Latinx-white disparities were smaller (− 0.00023; 95% CI − 0.00010, − 0.00036). Health disparities for the other measures demonstrate either an improvement of racial/ethnic and income disparities, or no statistically significant change in the trend (Appendix Figs. 11, 12, 13, 14, and 15).

## Discussion

From 1997 to 2018, health equity for children in the United States has generally improved. This result holds across several measures of physical and mental health. Although it cannot be known whether more progress was possible over the previous 20 years, the United States appeared to be moving in the right direction prior to the COVID-19 pandemic.

However, the reasons for this progress are less clear. A number of policies could be partially responsible, including expansions of insurance coverage, increases in tax credits for families with children, and greater access to preschool (Isaacs et al., 2019). Because the causes of health inequities are numerous and deeply rooted, disentangling any one of these policies from the others is challenging—and may not be necessary (Woolf, 2017). Future analyses could provide greater insight into the relationship between public policy and health equity by exploring the associations between the two at the state and sub-state level.

Behavioral and cultural changes outside the policy process may also contribute to improved health equity. Parental time spent caring for children has increased over the past 20 years, in part because of an increased understanding of the benefits to child development (Dotti Sani & Treas, 2016). Furthermore, improvements in knowledge have translated into better health behaviors and healthcare quality during pregnancy (Aizer & Currie, 2014). Wider recognition of the importance of population health may also play a role, as practitioners and policymakers realize that health equity is at stake in all forms of policy (Hall & Jacobson, 2018; Purnell et al., 2016).

A previous analysis found declining health equity among adults over the same period, a result that provides a noteworthy contrast to the findings here (Zimmerman & Anderson, 2019). However, upon closer examination of the age-stratified analysis, these findings appear more consistent with each other. Improvements in health equity appear to be limited to younger children, with gains for adolescents muted or non-existent. Taken together, the results suggest that society’s greater relative willingness to protect and invest in younger children may be buffering them against the social and economic forces that have led to reduced health equity among adults, while results for any such investments in adolescents are lagging.

Previous analysis found small and decreasing Black–white disparities among adults, but large and increasing income disparities (Zimmerman & Anderson, 2019). This analysis finds both types of disparities are narrowing among children. The contrast with trends in adult disparities may be somewhat explained by the political-economic structuring of public benefits, which have long been more generous for children and families than for adults (Moffitt, 2015).

However, the gains made in the past few decades are under threat. Prior to the current Presidential administration and Congress, projections on public spending for children over the next decade forecast a deep decline in federal outlays to health and other social services (Isaacs et al., 2019). Should that prediction come to pass, the most vulnerable children and families would be disproportionately affected. The child tax credit—passed by Congress as part of the American Rescue Plan—is a promising start, but more permanent forms of funding are needed to bolster the improvements in health equity documented here.

Furthermore, the COVID-19 pandemic threatens child health in deeply inequitable ways (Oronce et al., 2020). Although the current evidence indicates the broader child population is not highly susceptible to the most acute symptoms of the virus, evidence of racial/ethnic disparities in COVID-19-related hospitalizations and other syndromes among children is well-documented (Godfred-Cato et al., 2020; Kim et al., 2020). Furthermore, inequities in mental health outcomes are likely to be exacerbated by large-scale quarantining, social distancing, and other stressors (Ravens-Sieberer et al., 2021). Additionally, inequities in mortality and unemployment for older family members mean that less-privileged children could feel the ripples of the current crisis for years. Finally, school closures have caused deep and highly unequal harm to children’s well-being and development (Christakis et al., 2020; National Academies of Sciences & Medicine, 2020). To summarize, while many opportunities to promote health equity exist during times of relative prosperity, a complex crisis such as the COVID-19 pandemic jeopardizes health equity through many, indeed most, of the root causes of health.

This study has limitations. The NHIS is restrictive in both the years and measures available. Second, self-reported health is reweighted using a study consisting of primarily adults, plus adolescents aged 12 and over (Van Doorslaer & Jones, 2003). More reassuringly, several other international studies have found the weighting scheme to be consistent (Burström et al., 2014; Christiansen et al., 2018). We are unaware of any evidence to suggest parents would apply an alternative valuation to the different responses of their child’s health as compared to their own, but it remains plausible that a weighting scheme specifically estimated for children could produce different results. Finally, the health outcomes included in the NHIS are collected from the parent, rather than the child. While these measures have been carefully validated, they may not be as robust as direct observation by clinicians of child outcomes.

## Conclusion

Committing to health equity for current and future generations is an essential goal of population health, and doing so requires careful monitoring of progress using an objective measure of health equity distinct from health disparities. This analysis suggests some improvement over the past 2 decades in child health equity; public policy appears to be serving its function of creating ever-more equal opportunity. At the same time, progress can stall or even be reversed, indicating that more should be done to continue to improve health equity for the nation’s children. To protect the fragile gains in health equity among children, our nation must take systemic action to address persistent inequities and provide the conditions that will allow its children to reach their health potential.

## Figures and Tables

**Fig. 5:**
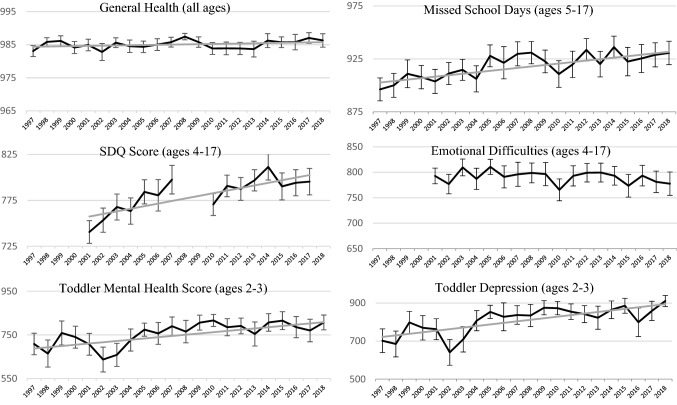
Health equity metric with 90th percentile benchmark, selected child health indicators: 1997–2018. Source: 1997–2018 National Health Interview Survey, Sample Child Questionnaire. HEM = Health Equity Metric. *SDQ* Strength and Difficulties Questionnaire. Age of respondents is shown in parentheses. The graphs display the value of the HEM for the entire child population over the study period for the various measures. Rather than using the median health of the privileged population within the age group as the benchmark level of health, this version uses the 90th percentile of the age group. HEM is rescaled by 1000 to assist with interpretability—a value of 1000 indicates perfect health equity. Response values in reweighted general health are based on the results from Van Doorslaer & Jones, 2003. 95% CI for the health equity metric estimates are denoted by hashes. Omitted points reflect the question is not included in the survey for that year. The median trend from Monte Carlo simulation is shown in gray if the 95% credible interval indicates the trend is significantly different from 0

**Fig. 6:**
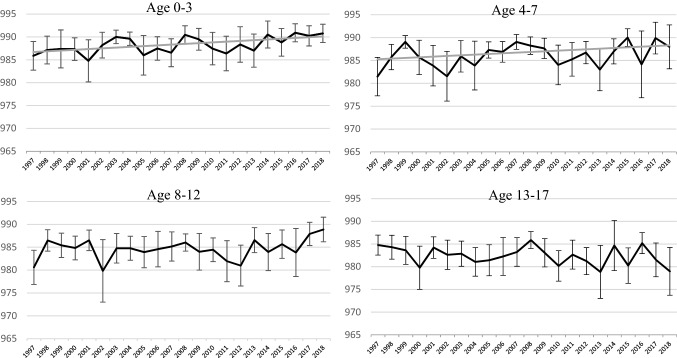
Health equity metric with 90th percentile benchmark, reweighted parent-reported health, by age group: 1997–2018. Source: 1997–2018 National Health Interview Survey, Sample Child Questionnaire. *HEM* Health Equity Metric. The graphs display the value of the health equity metric for the child population by age group over the study period. Rather than using the median health of the privileged population within the age group as the benchmark level of health, this version uses the 90th percentile of the age group. HEM is rescaled by 1000 to assist with interpretability—a value of 1000 indicates perfect health equity. Response values in reweighted general health are based on the results from Van Doorslaer & Jones, 2003. 95% CI for the health equity metric estimates are denoted by hashes. The median trend from Monte Carlo simulation is shown in gray if the 95% credible interval indicates the trend is significantly different from 0

**Fig. 7:**
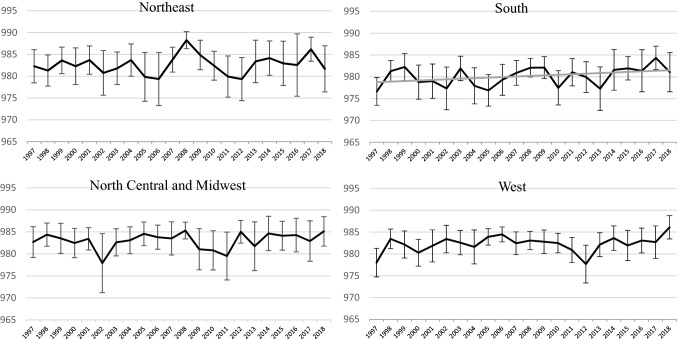
Health equity metric, reweighted parent-reported health, by census region: 1997–2018. Source: 1997–2018 National Health Interview Survey, Sample Child Questionnaire. *HEM* Health Equity Metric. The graphs display the value of the health equity metric for the child population by census region over the study period. HEM is rescaled by 1000 to assist with interpretability—a value of 1000 indicates perfect health equity. Response values in reweighted general health are based on the results from Van Doorslaer & Jones, 2003. 95% CI for the health equity metric estimates are denoted by hashes. The median trend from Monte Carlo simulation is shown in gray if the 95% credible interval indicates the trend is significantly different from 0

**Fig. 8:**
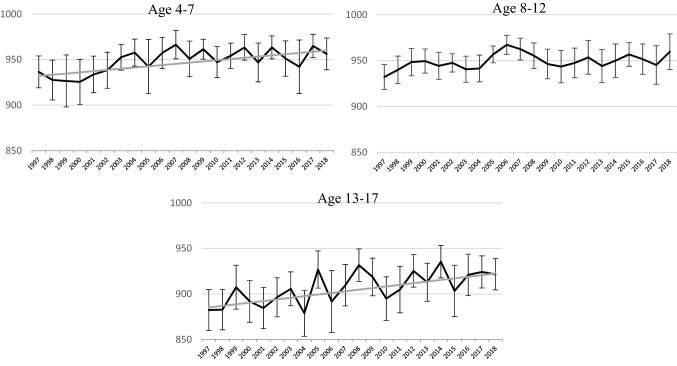
Health equity metric, school days missed due to illness or injury, by age group: 1997–2018. Source: 1997–2018 National Health Interview Survey, Sample Child Questionnaire. *HEM* Health Equity Metric. The graphs display the value of the HEM for the child population by age subgroup over the study period. HEM is rescaled by 1000 to assist with interpretability—a value of 1000 indicates perfect health equity. 95% CI for the health equity metric estimates are denoted by hashes. The median trend from Monte Carlo simulation is shown in gray if the 95% credible interval indicates the trend is significantly different from 0

**Fig. 9:**
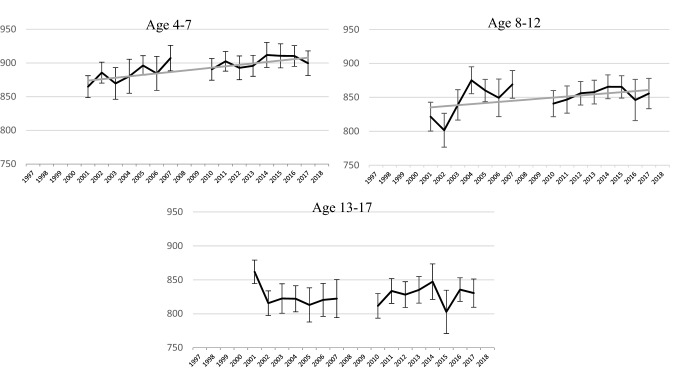
Health equity metric, strength and difficulties questionnaire score, by age group: 1997–2018. Source: 1997–2018 National Health Interview Survey, Sample Child Questionnaire. *HEM* Health Equity Metric. The graphs display the value of the HEM for the child population by age subgroup over the study period. HEM is rescaled by 1000 to assist with interpretability—a value of 1000 indicates perfect health equity. Omitted points reflect the question is not included in the survey for that year. 95% CI for the health equity metric estimates are denoted by hashes. The median trend from Monte Carlo simulation is shown in gray if the 95% credible interval indicates the trend is significantly different from 0

**Fig. 10:**
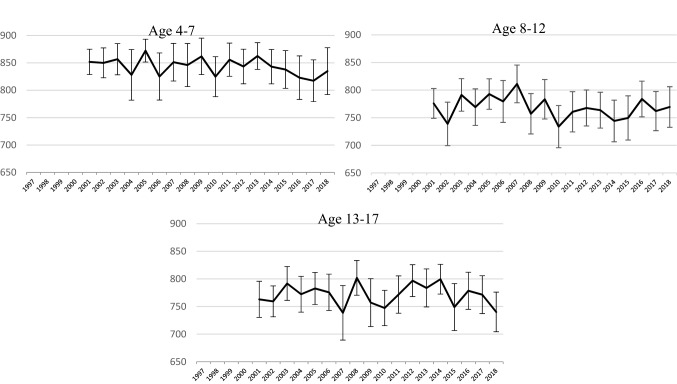
Health equity metric, emotional difficulties, by age group: 1997–2018. Source: 1997–2018 National Health Interview Survey, Sample Child Questionnaire. *HEM* Health Equity Metric. The graphs display the value of the HEM for the child population by age subgroup over the study period. HEM is rescaled by 1000 to assist with interpretability—a value of 1000 indicates perfect health equity. Omitted points reflect the question is not included in the survey for that year. 95% CI for the health equity metric estimates are denoted by hashes. The median trend from Monte Carlo simulation is shown in gray if the 95% credible interval indicates the trend is significantly different from 0

**Fig. 11:**
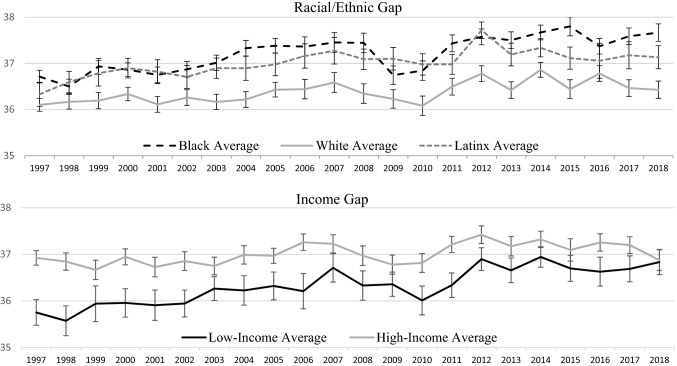
Disparities measures, school days missed due to illness or injury: 1997–2018. Source: 1997–2018 National Health Interview Survey, Sample Child Questionnaire. The trends here show the average value of the parent-reported school days missed due to health-related issues in the prior year for respective subpopulations. The measure is top-coded at 40 days, and reverse coded such that 0 is equal to 40 or more days missed and 40 is equal to no days missed. Low-income and high-income are defined as living in a household with income < 150% of the federal poverty line and living in a household with income ≥ 400% of the federal poverty line, respectively. 95% CI for the annual estimates are denoted by hashes. Monte Carlo simulation estimates show that the advantage for black children increased (0.024; 95% CI: 0.011, 0.036) and the income gap narrowed (− 0.033; 95% CI: − 0.022, − 0.045) over the study period. The increase in the Latinx advantage over whites is not statistically significant (0.0085; 95% CI: − 0.0019, 0.0190)

**Fig. 12:**
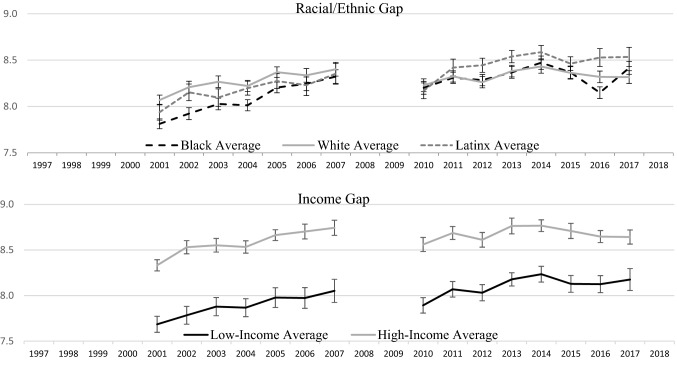
Disparities measures, strength and difficulties questionnaire score: 1997–2018. Source: 1997–2018 National Health Interview Survey, Sample Child Questionnaire. The trends here show the average value of the parent-reported Strength and Difficulties Questionnaire measure for respective subpopulations. Omitted points reflect the question is not included in the survey for that year. Low-income and high-income are defined as living in a household with income < 150% of the federal poverty line and living in a household with income ≥ 400% of the federal poverty line, respectively. 95% CI for the annual estimates are denoted by hashes. Monte Carlo simulation estimates show that the black-white gap (− 0.019; 95% CI: − 0.011, − 0.026), the Latinx-white gap (− 0.023; 95% CI: − 0.017, − 0.028), and the income gap (− 0.013; 95% CI: − 0.006, − 0.019) narrowed over the study period

**Fig. 13:**
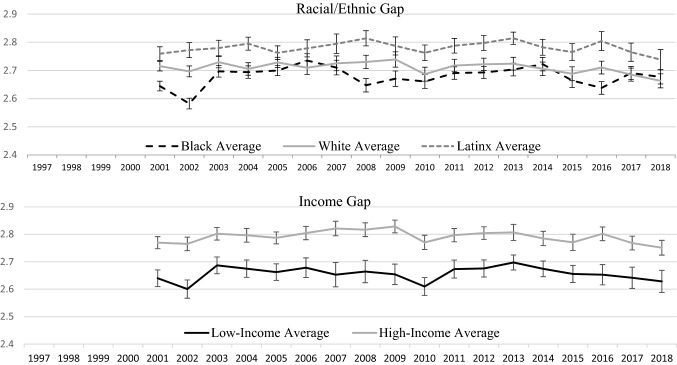
Disparities measures, emotional difficulties: 1997–2018. Source: 1997–2018 National Health Interview Survey, Sample Child Questionnaire. The trends here show the average value of the parent-reported emotional difficulties measure for respective subpopulations. Omitted points reflect the question is not included in the survey for that year. Low-income and high-income are defined as living in a household with income < 150% of the federal poverty line and living in a household with income ≥ 400% of the federal poverty line, respectively. 95% CI for the annual estimates are denoted by hashes. Monte Carlo simulation estimates show that the black–white gap narrowed (− 0.0029; 95% CI: − 0.0005, − 0.0052) over the study period. The increase in the Latinx advantage over whites is not statistically significant (0.0016; 95% CI: − 0.0001, 0.0033) and the negative trend in the income gap is not statistically significant (− 0.0009; 95% CI: − 0.0028, 0.0010)

**Fig. 14:**
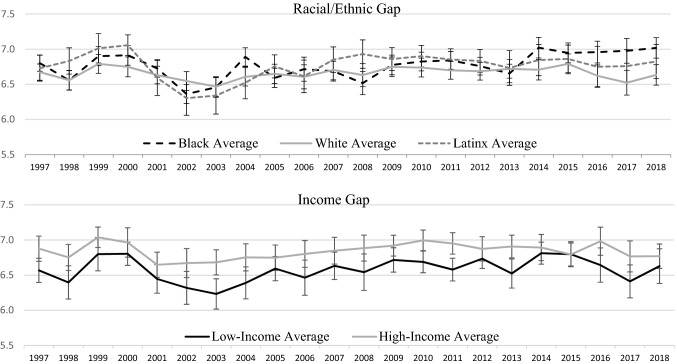
Disparities measures, toddler mental health indicator: 1997–2018. Source: 1997–2018 National Health Interview Survey, Sample Child Questionnaire. The trends here show the average value of the parent-reported toddler mental health indicator for respective subpopulations. Low-income and high-income are defined as living in a household with income < 150% of the federal poverty line and living in a household with income ≥ 400% of the federal poverty line, respectively. 95% CI for the annual estimates are denoted by hashes. Monte Carlo simulation estimates show that the black–white gap narrowed (− 0.012; 95% CI: − 0.002, − 0.023) over the study period. The increase in the Latinx advantage over whites is not statistically significant (− 0.004; 95% CI: 0.005, − 0.013) and the negative trend in the income gap is not statistically significant (− 0.004; 95% CI: − 0.014, 0.005)

**Fig. 15:**
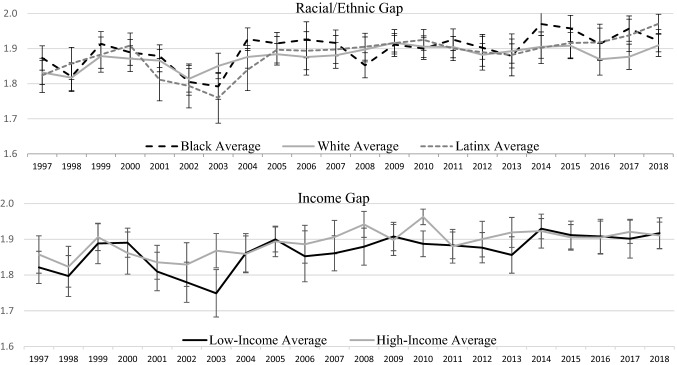
Disparities measures, toddler depression: 1997–2018. Source: 1997–2018 National Health Interview Survey, Sample Child Questionnaire. The trends here show the average value of the parent-reported toddler depression measure for respective subpopulations. Low-income and high-income are defined as living in a household with income < 150% of the federal poverty line and living in a household with income ≥ 400% of the federal poverty line, respectively. 95% CI for the annual estimates are denoted by hashes. Monte Carlo simulation estimates show that the negative trend in neither the black–white gap (− 0.0012; 95% CI: − 0.0037, 0.0012) nor the income gap (– 0.0010; 95% CI: − 0.0033, 0.0012) is statistically significant over the study period. However, the improvement in Latinx over the white population is statistically significant (− 0.0023; 95% CI: − 0.0003, − 0.0042)

## Data Availability

Original data is available publicly on the IPUMS website.

## Appendix

See Figs. 5, 6, 7, 8, 9, 10, 11, 12, 13, 14, 15.

## Code Availability

STATA code for the analysis can be provided upon request.
